# Unusual Cardiac Presentation of COVID-19 and Use of Convalescent Plasma

**DOI:** 10.1155/2020/8863195

**Published:** 2020-10-02

**Authors:** William R. Hartman, Aaron S. Hess, Joseph P. Connor

**Affiliations:** ^1^Department of Anesthesiology, University of Wisconsin-Madison School of Medicine and Public Health, USA; ^2^Department Pathology and Laboratory Medicine, University of Wisconsin-Madison School of Medicine and Public Health, USA

## Abstract

COVID-19 infection caused by the SARS-CoV-2 virus has been associated with cardiac abnormalities, including conduction abnormalities. Convalescent plasma is emerging as a potentially safe and effective treatment option for patients severely or critically ill with COVID-19. Here, we describe a case of a COVID-19 patient with new-onset cardiac ectopy who had near resolution of his cardiac sequelae following convalescent plasma transfusion.

## 1. History of Presentation

A 62-year-old man with history of moderate persistent asthma, sinus bradycardia, COPD, and chronic pain was admitted to a tertiary health care center with laboratory-confirmed COVID-19 after 5 days of increased cough and shortness of breath. He had been taking 20 mg of prednisone daily for four days prior to admission as well as three doses of an old prescription of amoxicillin-clavulanate. He had not taken any hydroxychloroquine. Proper ethical guidelines have been followed, and the patient has documented consent to be part of a research study as well as have his case used in future publications.

## 2. Differential Diagnosis

- COPD Exacerbation
- Asthma Exacerbation
- Bacterial pneumonia
- COVID-19 pneumonia.

## 3. Investigations

**Figure 1:**
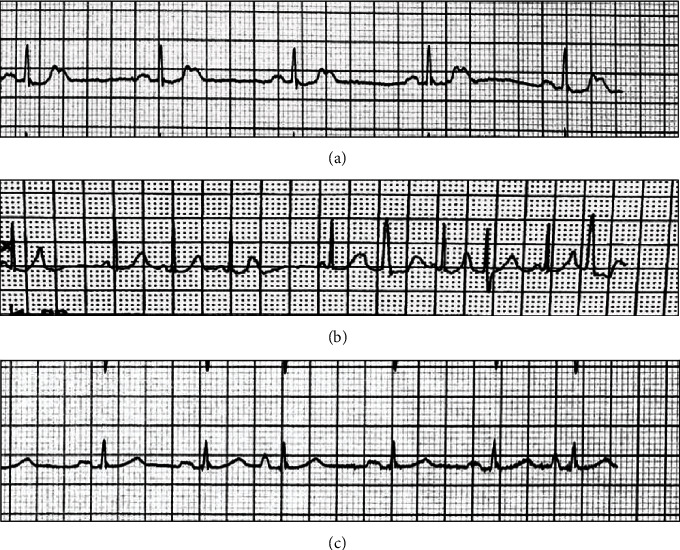
Representative lead II ECG tracings. ECG from day 1 (a), prior to convalescent plasma treatment (b), and after convalescent plasma treatment (c). The ectopy that presented during hospitalization was nearly resolved following treatment with convalescent plasma.

His admission D-dimer was 0.73 mcg/mL, and his C-reactive protein (CRP) was 0.8 mg/dL. His baseline heart rate was between 40 and 50 bpm but fell as low as 30 bpm overnight. His SpO2 remained >92% on room air. He was admitted for monitoring and remained clinically stable until hospital day 2 when he developed new tachycardia, worsening cough, and shortness of breath. His telemetry revealed frequent premature atrial contractions (PACs) as well as new premature ventricular contractions (PVCs) and apparent trigeminy (Figure 1). He was placed on 2 L/min oxygen by nasal cannula. His troponin was negative. Repeat D-dimer was 1.35 mcg/mL, CRP was 5.7 mg/dL, ferritin was 188, and potassium was 4.1 mEq/L.

## 4. Management

Based on his sudden deterioration, this patient was consented for and received 217 mL of COVID-19 convalescent plasma. No other interventions were made at the time. 24 hours later, his heart rate had improved to the 60-70 bpm with less frequent PACs and PVCs and he was breathing comfortably on room air. His CRP decreased to 5.3 mg/dL and ferritin to 168. He was able to pass a 6-minute walk test with saturations of 94% and greater, and he competed evaluations with occupational and physical therapy. 36 hours after transfusion, the patient was discharged from the hospital reporting that he felt improved, although still with cough and subjective shortness of breath with activity similar to his baseline COPD symptoms.

## 5. Discussion

At the end of 2019, a novel coronavirus was identified as the cause of a cluster of pneumonia cases in Wuhan, China. The virus rapidly spread, resulting in a global pandemic with a devastating impact on populations and the global economy. While most infections remain asymptomatic or recover as outpatients from milder variations of the disease, a subset of individuals are hospitalized with some requiring intensive care unit admission both with or without mechanical ventilation. Although fever and respiratory symptoms are the most common manifestations of disease in admitted patients, some patients present with chest palpitations or other cardiac-related symptoms including chest pain. Patients presenting with cardiac-related symptoms have been estimated to be approximately 7% of cases [1]. In Wuhan, China, abnormal cardiac conduction/arrhythmia was seen in 17% of hospitalized patients in general care but was seen in twice as many (44%) of the patients require intensive care unit admission [2]. COVID-19 has been associated with cardiac morbidity and mortality, with individuals having evidence of cardiac damage as defined by an increased troponin level having a worse outcome than those without elevated troponin [3].

In a study of myocardial injury in COVID-19 patients in Wuhan, China, patients with myocardial injury had higher leukocyte (*P* < .001), lower lymphocyte (*P* < .001), and lower platelet counts (*P* < .001) [4]. A retrospective study from another Wuhan hospital demonstrated that patients with high troponin-T levels had leukocytosis (*P* < .001), increased neutrophils (*P* < .001), and decreased lymphocytes (*P* = .01) [5]. Our patient had an elevated leukocyte count but a normal lymphocyte count and elevated neutrophils. While his troponin-T level was not elevated, he did have an elevated creatinine kinase and D-dimer.

The pathophysiology of COVID-19-associated cardiac disease and the conduction abnormalities described is complex; however, it is thought to most likely be secondary to excessive systemic inflammation leading to myocardial damage [6]. Previous reports of COVID-19-associated acute myocardial injury suggest that cardiovascular sequalae are secondary to a high systemic inflammatory response and the associated excessive cytokine release [7, 8]. Other contributing factors that have been reported include the presence of preexisting cardiac disease, primary cardiac myocyte damage by direct viral infection, and the increased and often overwhelming demand brought on by hypoxia associated with severe infection and respiratory failure [9].

Cardiac conduction abnormalities were also observed during the original SARS-CoV outbreak (2002–2003). *In vitro* studies demonstrated that the angiotensin-converting enzyme 2 (ACE2) was responsible for viral uptake into host cells via spike protein and that the ACE2 receptor is abundant in cardiac myocytes [10]. Autopsy studies of mice and rabbits infected with SARS-CoV demonstrated a direct viral RNA inclusion in myocytes of specimens with cardiac injury and conduction system disease [11]. ACE2 is also the viral receptor for SARS-CoV2 [12].

Convalescent plasma (CP) is an emerging treatment for COVID-19 infection, and early data suggests that it is safe. Convalescent plasma is the liquid part of blood containing antibodies and is collected from individuals who have recovered from a viral infection. It has been used for over 100 years in treatment of other viral pandemics in which no vaccine was available including the “Spanish” influenza, SARS, MERS, Argentine hemorrhagic fever, and Ebola. The results from using convalescent plasma as a treatment for these diseases remain somewhat uncertain. While convalescent plasma has become recognized as the treatment of choice for Argentine hemorrhagic fever, convalescent plasma use in Ebola, while safe, has not proven efficacious. Published reports of convalescent plasma use in other coronaviruses including MARS and SERS are limited to case reports and case series or retrospective nonrandomized analyses which are difficult to draw conclusions from [13]. An early randomized study of convalescent plasma in COVID-19 patients was underpowered and terminated early, but failed to establish effectiveness as a therapy for COVID-19 disease [14]. Convalescent plasma is currently being studied as a potential therapy for hospitalized patients suffering from COVID-19 infection [15].

CP has not been reported as a treatment for patients with cardiac symptoms. We present the case of a man admitted with COVID-19 who developed severe worsening of his baseline arrythmia and recovered after CP administration [16]. Here, we report the case of a 62-year-old male with COVID-19-associated arrythmias that improved following administration of COVID-19 convalescent plasma. Of particular interest is that the abnormal conduction episodes documented were not in the setting of hydroxychloroquine administration. This patient developed palpitations associated with new-onset PACs and PVCs. There were laboratory markers of severe disease, but not myocardial injury. Following convalescent plasma administration, he had rapid improvement in his symptoms and electrocardiogram findings and was discharged shortly thereafter.

We do not know by what mechanism convalescent plasma might have aborted this patient's ectopy and led to clinical improvement. It is possible that donor anti-SARS-CoV-2 antibodies may have contributed to rapid inactivation of viral particles and so reduced myocardial irritation. Although this is only a hypothesis to explain the temporal association between treatment and improvement, no other medications were administered concurrently, and he only was hydrated with oral water and electrolyte drinks. This represents the first reported case of COVID-19-associated cardiac ectopy being treated and apparently terminated after administration of COVID-19 convalescent plasma. While purely speculative, we postulate that convalescent plasma might be used effectively in infected individuals who display signs of cardiac conduction system abnormalities and not only those with primary pulmonary presentations.

## 6. Conclusion

COVID-19 has been associated with cardiac conduction system abnormalities. Patients with confirmed COVID-19 should be monitored closely for changes from their cardiac baseline including new-onset PACs and PVCs. Potential viral mechanisms for conduction system abnormalities include myocardial inflammation or direct viral infiltration. Convalescent plasma, an emerging treatment for COVID-19 infection, should be considered as an early therapy when patients present with symptoms of myocardial irritability. Further studies are clearly needed to help delineate the safety and effectiveness of this treatment.

## 7. Learning Objective

COVID-19 manifests itself in a variety of ways in different patients. A less common, but serious complication of this virus is new cardiac conduction abnormalities. With no cure for the disease, Convalescent plasma was attempted as a way to neutralize the virus and quell the cardiac ectopy. In this patient, convalescent plasma therapy was able to return him to his baseline.

## References

[1] Glantz S. A. (1993). It is all in the numbers. *Journal of the American College of Cardiology*.

[2] Zhou F., Yu T., du R. (2020). Clinical course and risk factors for mortality of adult inpatients with COVID-19 in Wuhan, China: a retrospective cohort study. *Lancet*.

[3] Wang W., Xu Y., Gao R. (2020). Detection of SARS-CoV-2 in different types of clinical specimens. *JAMA*.

[4] Sullivan H. C., Roback J. (2020). Convalescent plasma: therapeutic hope or hopeless strategy in the SARS-CoV-2 pandemic. *Transfusion Medicine Reviews*.

[5] Shi S., Qin M., Shen B. (2020). Association of cardiac injury with mortality in hospitalized patients with COVID-19 in Wuhan, China. *JAMA Cardiology*.

[6] Lippi G., Lavie C. J., Sanchis-Gomar F. (2020). Cardiac troponin I in patients with coronavirus disease 2019 (COVID-19): evidence from a meta-analysis. *Progress in Cardiovascular Diseases*.

[7] Driggin E., Madhavan M. V., Bikdeli B. (2020). Cardiovascular considerations for patients, health care workers, and health systems during the coronavirus disease 2019 (COVID-19) pandemic. *Journal of the American College of Cardiology*.

[8] Kochi A. N., Tagliari A. P., Forleo G. B., Fassini G. M., Tondo C. (2020). Cardiac and arrhythmic complications in patients with Covid-19. *Journal of Cardiovascular Electrophysiology*.

[9] Wang W., Ye L., Ye L. (2007). Up-regulation of IL-6 and TNF-alpha induced by SARS-coronavirus spike protein in murine macrophages via NF-kappaB pathway. *Virus Research*.

[10] Cheng P., Zhu H., Witteles R. M. (2020). Cardiovascular risks in patients with COVID-19: potential mechanisms and areas of uncertainty. *Current Cardiology Reports*.

[11] Zheng Y. Y., Ma Y. T., Zhang J. Y., Xie X. (2020). COVID-19 and the cardiovascular system. *Nature Reviews Cardiology*.

[12] Alexander L. K., Keene B. W., Small J. D., Yount B Jr, Baric R. S. (1994). Electrocardiographic changes following rabbit coronavirus-induced myocarditis and dilated cardiomyopathy. *Coronaviruses*.

[13] Oudit G. Y., Kassiri Z., Jiang C. (2009). SARS-coronavirus modulation of myocardial ACE2 expression and inflammation in patients with SARS. *European Journal of Clinical Investiagtion*.

[14] Donoghue M., Wakimoto H., Maguire C. T. (2003). Heart block, ventricular tachycardia, and sudden death in ACE2 transgenic mice with downregulated connexins. *Journal of Molecullar and Cellular Cardiology*.

[15] Li L., Zhang W., Hu Y. (2020). Effect of convalescent plasma therapy on time to clinical improvement in patients with severe and life-threatening COVID-19: a randomized clinical trial. *JAMA*.

[16] Guo T., Fan Y., Chen M. (2020). Cardiovascular implications of fatal outcomes of patients with coronavirus disease 2019 (COVID-19). *JAMA Cardiology*.

